# A dual pollination strategy combining perfume attraction and food deception in *Galeandra blanchetii* E.S. Rand (Orchidaceae)

**DOI:** 10.1111/plb.70288

**Published:** 2026-09-25

**Authors:** D. M. Camara, A. W. C. Ferreira, A. P. S. Caetano, A. Domingos‐Melo, S. Albuquerque‐Lima, P. Milet‐Pinheiro, I. C. Machado, E. M. Pessoa

**Affiliations:** ^1^ Programa de Pós‐Graduação em Biodiversidade, Ambiente e Saúde Universidade Estadual do Maranhão Caxias Brazil; ^2^ Departamento de Biologia, Laboratório de Estudos de Orquídeas, Cidade Universitária Dom Delgado Universidade Federal do Maranhão São Luís Brazil; ^3^ Departamento de Botânica e Ecologia, Laboratório de Estudos Integrados de Plantas Universidade Federal de Mato Grosso Cuiabá Brazil; ^4^ Departamento de Biociências, Laboratório de Biologia Floral e Ecologia Reprodutiva Universidade Federal de Sergipe Itabaiana Brazil; ^5^ Instituto de Biodiversidade e Sustentabilidade—NUPEM Universidade Federal do Rio de Janeiro Rio de Janeiro Brazil; ^6^ Colegiado de Biologia, Laboratório de Interações Ecológicas e Semioquímicos Universidade de Pernambuco Petrolina Brazil; ^7^ Programa de Pós‐Graduação em Biologia Vegetal, Departamento de Botânica Universidade Federal de Pernambuco Recife Brazil; ^8^ Universidade Federal do ABC Centro de Ciências Naturais e Humanas Santo André São Paulo Brazil

**Keywords:** Catasetinae, Euglossini, floral scent, osmophores, pollination

## Abstract

- The evolutionary origin of the perfume flower syndrome remains unclear. Orchids of the subtribe Catasetinae help elucidate the evolution of this syndrome, but knowledge gaps regarding the pollination biology of *Galeandra* hinder a full understanding of the process. This study addresses this gap by investigating *G. blanchetii*, an epiphytic Catasetinae species endemic to Brazil. We explored its floral and reproductive biology, including floral anatomy, scent characterization and pollinator mechanical fit, to clarify the role of floral signals in pollinator attraction.
- We found epidermal osmophores and unicellular glandular trichomes in the labellum, but no nectar production was detected. Floral scent comprised 34 compounds, with methyl salicylate and methyl benzoate as the most abundant constituents. Despite being self‐compatible, *G. blanchetii* requires biotic vectors for reproduction.
- The flowers attracted both male and female bees; however, only some male Euglossini bees exhibited fragrance‐collecting behaviour, whereas females were observed only searching for nectar. Pollinaria were removed only when bees entered the spur while searching for nectar, indicating that fragrance collection alone did not result in pollination.
- We conclude that *G. blanchetii* exhibits a dual pollination strategy combining perfume attraction and food deception. Floral fragrance provides a resource for male Euglossini bees, whereas melittophilous floral traits and the absence of nectar are consistent with food deception in floral visitors searching for nectar. This combination provides further insights into the diversity and evolution of pollination strategies within *Galeandra* and, more broadly, within Catasetinae.

## Graphical abstract

*Galeandra blanchetii* exhibits a dual‐attraction strategy: male euglossine bees are lured by floral scents as a resource, while both sexes are deceived by non‐existent nectar. Pollination only occurs when bees enter the spur to search for nectar, highlighting an evolutionary transition towards the perfume flower syndrome in Catasetinae.

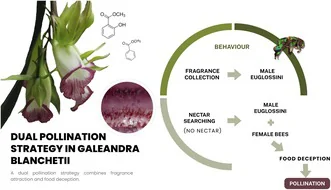

## INTRODUCTION

Interactions between plants and pollinators are mediated by floral signals, including visual and olfactory cues, which influence pollinator orientation and foraging behaviour (Rech *et al*. 2014; Araújo *et al*. 2024). These signals may act in an integrated and context‐dependent manner, modulating pollinator responses and consequently floral detection (Raguso 2004). Floral signals are frequently associated with the presence of rewards such as nectar, pollen, lipids or fragrances, which play a central role in the reproductive success of angiosperms by promoting floral visitation, pollination success and fertilization efficiency (Johnson *et al*. 2003; Blaauw & Isaacs 2014; Rech *et al*. 2014; Makino & Ohashi 2017).

In Orchidaceae, these interactions reach a remarkable degree of specialization, involving both food rewards, such as nectar, pollen and edible trichomes (pseudopollen), and non‐food rewards, including oils, waxes, resins and volatile compounds used by pollinators in specific behavioural contexts (Dressler 1982; Davies *et al*. 2003, Davies & Turner 2004; Nunes *et al*. 2017; Gomiz *et al*. 2017; Phillips *et al*. 2020; Reposi *et al*. 2021; Milet‐Pinheiro *et al*. 2022; Ackerman *et al*. 2023). In contrast, a substantial proportion of orchid species adopt deceptive pollination strategies, estimated to occur in approximately 46% of the species for which pollination data are available, in which floral signals mimic the presence of rewards without providing an effective resource (Ackerman 1986; Schiestl 2005; Jersáková *et al*. 2006; Ackerman *et al*. 2023).

Within the subtribe Catasetinae (Epidendroideae), this diversity of reproductive strategies is particularly pronounced, making the group a relevant model for investigating the evolution of pollination syndromes (Pérez‐Escobar *et al*. 2016; Pansarin & Maciel 2017). This subtribe occurs throughout the tropical and subtropical Americas and comprises eight genera (Romero & Pridgeon 2009; Batista *et al*. 2014). In most representatives, pollinator interactions are primarily mediated by floral fragrances collected by male Euglossini bees, which act as effective pollinators (Dodson 1962; Dressler 1982; Williams & Whitten 1983; Nunes *et al*. 2017). These volatile compounds are stored in specialized structures on the hind legs of the bees and subsequently used in courtship displays, playing an important role in female attraction and male reproductive success (Eltz *et al*. 1999; Bisrat & Jung 2022; Henske *et al*. 2023; Ayasse 2023). Other reward types also occur within the subtribe, including edible trichomes and floral oils, although these are comparatively less common (Pansarin *et al*. 2009; Pansarin & Maciel 2017).

Phylogenetic analyses based on Bayesian inference have revealed a consistent evolutionary structure within Catasetinae (Pansarin & Maciel 2017). In these studies, the clade formed by *Cyanaeorchis* and *Grobya*, both associated with food rewards, emerged as sister to the remaining lineages of the subtribe, whereas a large euglossinophilous clade characterized by fragrance production included most of the remaining genera, such as *Catasetum*, *Cycnoches* and *Mormodes*. *Galeandra* occupied a distinct phylogenetic position within Catasetinae, as sister to the *Grobya* and *Cyanaeorchis* clade rather than being directly embedded within the principal euglossinophilous lineage. In addition to this phylogenetic placement, the genus exhibits marked floral variation, particularly in spur morphology, with several species presenting elongated spurs commonly associated with specialized interactions with bees (Martins *et al*. 2018). Together, these characteristics highlight the potential of *Galeandra* to provide new insights into the evolution of floral rewards, pollinator attraction and pollination strategies within the subtribe.

Despite this evolutionary relevance, the reproductive biology of *Galeandra* remains poorly understood, particularly regarding pollinator attraction mechanisms and the presence or absence of floral rewards. Therefore, in this study, we integrated multiple approaches to investigate the floral and reproductive biology of *Galeandra blanchetii* E.S. Rand, a species endemic to Brazil. Specifically, we asked: (1) Do flowers of *G. blanchetii* provide rewards? (2) If so, which type of reward is present? (3) If floral scents are produced, what is the composition of the floral bouquet? (4) Which visitors act as effective pollinators? and (5) To what extent does reproduction depend on pollinators? We predicted that *G. blanchetii* may produce floral fragrances as a potential attractant while also exhibiting traits associated with deceptive pollination. By addressing these questions, we aimed to broaden our current understanding of the functional diversity of pollination systems within Catasetinae.

## MATERIALS AND METHODS

### Study species and location

*Galeandra blanchetii* is an epiphytic herb (Fig. 1) endemic to Brazil, occurring across the Amazon, Caatinga and Cerrado phytogeographical domains (Monteiro *et al*. 2010; Santos & Silva 2020; Monteiro 2024). This study was conducted in two areas of Amazonian forest within the municipality of São Luís, Maranhão State, northeastern Brazil. The first site was located at the Federal University of Maranhão (UFMA), Dom Delgado campus (approximately 2°33′32″ S, 44°18′35″ W). This 100‐hectare area comprises five urban forest fragments (Costa & Almeida 2020) as well as adjacent garden areas associated with these fragments (Costa *et al*. 2017). The second site is located approximately 16 km from the first, within the Environmental Protection Area of Itapiracó (APA Itapiracó) (2°31′37.48″ S, 44°12′46.3″ W).

**Fig. 1:**
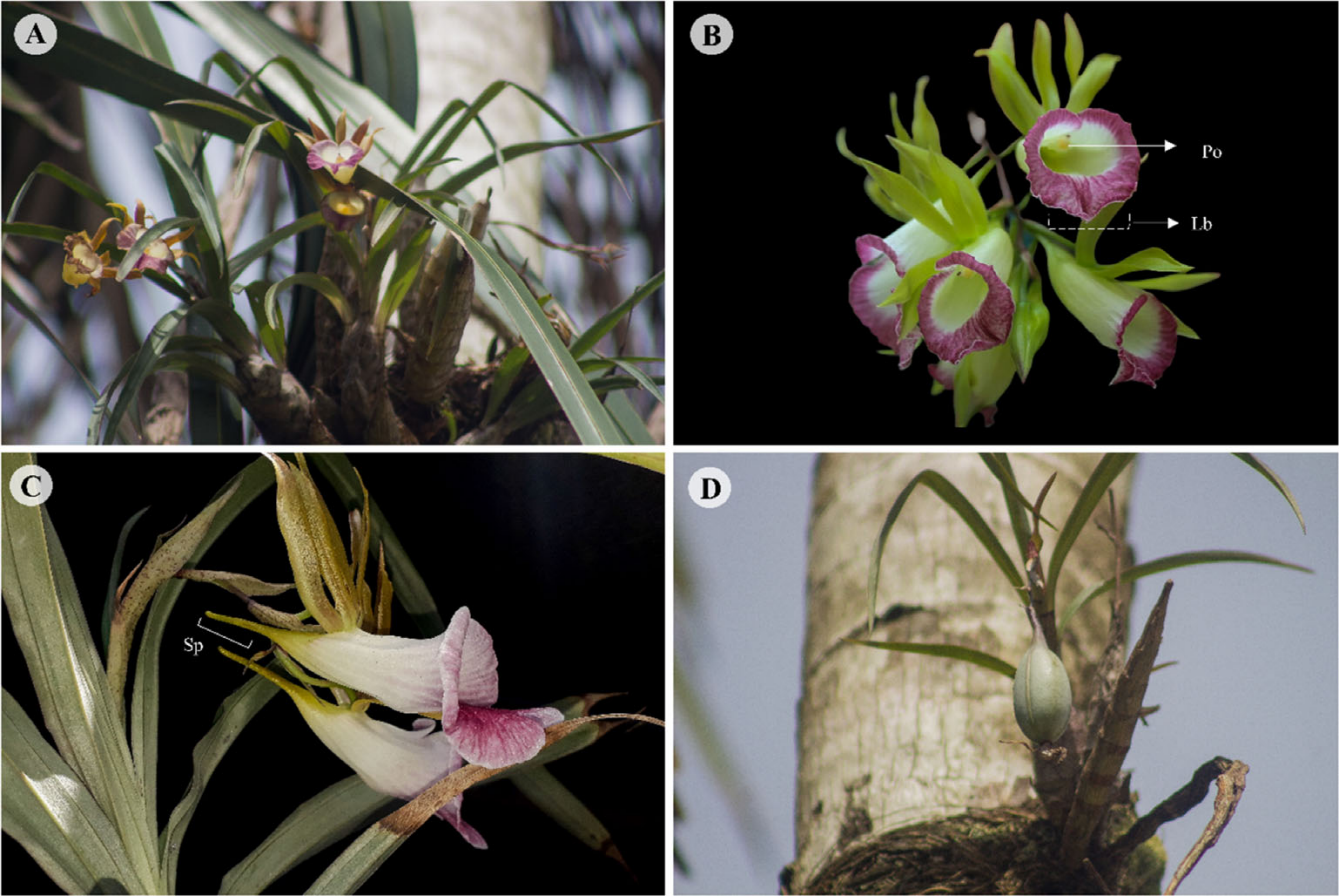
Epiphytical habit of *Galeandra blanchetii* (Orchidaceae) (A); Racemose inflorescence with flowers with vinaceous labellum in frontal view (B); Lb: Labellum, Po: Pollinarium; Inflorescence in lateral view, highlighting the tubular shape of the flower (C); Sp: spur; Immature capsule fruit (D).

According to the climatic classification system, the region has an Aw climate, characterized by a marked dry season in winter and a rainy season in summer (Köppen 1948). In the study area, the rainy season occurs from January to June and the dry season from July to December (Fig. S2). Mean annual precipitation is 2,117 mm and mean annual temperature is 26.7 °C (INMET – ‘Dados Meteorológicos: Estações Convencionais’; www.inmet.gov.br).

### Floral anthesis and stigma receptivity

Floral anthesis was monitored from the onset of floral opening until senescence in intact flowers (N = 30, 10 individuals) to determine whether the stigma was receptive from the first day of anthesis, indicating that flowers were available to potential pollinators at the beginning of the floral lifespan. In addition, we assessed whether pollinaria removal immediately triggered floral senescence. Stigmatic receptivity was evaluated in five flowers bearing pollinaria from five different individuals on the first day of anthesis by applying a drop of 3% hydrogen peroxide to the stigmatic surface. A positive indication of stigmatic receptivity was determined by the formation of bubbles on the surface where the peroxide was applied (Kearns & Inouye 1993).

### Morphology and histochemical tests

We analysed 30 flowers from 10 individuals under a binocular stereomicroscope. Measurements were taken using a digital pachymeter (MTX‐316119). For morphological study, the shape and size of floral parts were recorded. For histochemical tests, fresh flowers (n = 10, N = 5 individuals) were immersed in 0.1% (w/v) aqueous neutral red for 20 min to locate metabolically active areas, such as osmophores. After staining, the flowers were washed in flowing water and examined. To characterize the anatomical structure of secretory areas, flowers were manually sectioned. Following that, histochemical procedures were carried out to identify the main chemical compound classes present in the flowers: ruthenium red to detect mucilage (Gregory & Baas 1989); Sudan IV for total lipid detection (Pearse 1980); ferric chloride for phenolic compounds (Johansen 1940); Lugol for starch detection (Jensen 1962), NADI reagent for terpene detection (David & Carde 1964); Coomassie brilliant blue for protein detection (Fisher 1968); and periodic acid–Schiff stain (PAS) for water‐insoluble polysaccharides (Feder & O'brien 1968). Control histological sections were simultaneously prepared to the histochemical tests (sections mounted only in glycerine or water) to verify the natural aspect of the secretion coloration or untreated tissue (Pansarin *et al*. 2009).

### Floral visitors and pollination mechanism

Field observations were carried out during the day (6:00 to 17:00) during the flowering months from 2018 to 2023 to analyse the pollination process, visitation frequency and to capture floral visitors for subsequent identification. Individuals of *G. blanchetii* were monitored in both study areas for 70 days, totalling 515 h of observation (for field observation details, see Table S1). To exclude the possibility of nocturnal pollination, newly opened flowers of *G. blanchetii* with intact pollinia and without deposited pollinia were monitored over a 30‐day period (n = 20 flowers from 10 individuals). The flowers were marked at the end of the afternoon (17:00) and examined the following morning, around 7:00, to verify possible nocturnal pollination events.

In general, photographs and videos allowed us to distinguish between floral visitors and potential pollinators based on their behaviour. Floral visitors were classified as pollinators only when they were observed removing and depositing pollinaria (Ackerman *et al*. 2023). Those that only removed pollinaria, but were not observed depositing them on the stigma, were considered potential pollinators (Kephart 1983). The remaining visitors observed on flowers without any direct interaction with the pollinaria or stigma were considered only floral visitors. Visitors and potential pollinators were captured, identified (including sex determination) and photographed.

### Mechanical fit between bees and flowers

The following measures were taken from potential pollinators: total body length, height and width of the mesosome and proboscis (glossa) length. In tubular flowers, the distance between the column and the labellum plays an important role in pollinator adjustment (Watteyn *et al*. 2022, 2023). Thus, 30 flowers (N = 15 per population) were evaluated for the height from the labellum up to the rostellum cup (CN) and the height of the labellum up to the mean anther region (AN) (Fig. S3). Measurements of bees and flower structures were compared with boxplots.

### Floral scent collection

Samples of floral scents were collected using dynamic headspace methods (Milet‐Pinheiro *et al*. 2015; Brandt *et al*. 2020), aiming to chemically characterize the aromatic bouquet. Solitary flowers or those in inflorescences (34 flowers, N = 18 individuals from the locality in UFMA) were wrapped together in polyethylene bags. After accumulation of volatiles for 20 min, the scented air was drawn through an adsorbent filter for 20 min (quartz microvials ChromatoProbe); 15 mm × 2 mm diameter; containing 1.5 mg of Tenax‐TA (60–80 mesh, Supelco, Bellefonte, PA, USA) and 1.5 mg of Carbotrap (20–40 mesh, Supelco, Bellefonte, PA, USA), connected to a diaphragm pump (G12/01 EB, Rietschle Thomas, Puchheim, Germany) with silicone tubes.

The pump operated at a constant flow rate of 200 mL min^−1^, which was measured using an air flowmeter (0.1–1 L min^−1^). All samples (n = 8) were collected in the morning, from 9:00 to 11:00 a.m, which is the period with higher emissions of scents perceived by the human nose. To detect environmental contaminants, control samples (empty bags) were simultaneously collected at a distance of ca. 2 m from the target inflorescence using the same methods described above (n = 4 samples) and the same protocol described above for floral structures. This procedure allowed the exclusion of environmental contaminants.

### Chemical analysis of floral scents

Floral scent headspace samples were analysed using a gas chromatography–mass spectrometry (GC–MS) system (Agilent 7890A gas chromatograph and Agilent 5975C mass spectrometer, MSD series; Agilent Technologies, Palo Alto, CA, USA), equipped with a non‐polar HP‐5MS column (Agilent J&W; 30 m × 0.25 mm i.d., with a 0.25 μm film thickness) and a thermal separation probe (TSP, Agilent Technologies). Thermal desorption samples were loaded into the TSP, which was then inserted into the modified GC injector. The injector was operated in split mode (1:1) at a temperature of 250 °C. The GC oven temperature was held at 40 °C for 2 min and then increased at a rate of 4 °C min^−1^ until reaching a final temperature of 230 °C, which was maintained for 5 min. Electronic flow control was used to maintain a constant helium carrier gas flow rate of 1.0 mL min^−1^. The temperatures of the MS source and quadrupole were set at 230 and 150 °C, respectively. Mass spectra were obtained at 70 eV in electron ionization mode, with a scan range from 35 to 350 m/z. Compounds were identified by comparison of their mass spectra and retention indices with those available in the literature and in the MassFinder 4, NIST 11 and Wiley Registry 9th Edition databases (Adams 2007), using Agilent MSD Productivity ChemStation software (Agilent Technologies, Palo Alto, CA, USA). Whenever available, the identities of the compounds were verified by comparison of their retention times and mass spectra with those of authentic standards. The peak areas of the chromatograms were integrated to obtain the total ion current (TIC) signal, which was used to determine the relative percentage of each compound. Compounds detected in samples from the surrounding environment were excluded from the floral VOC (volatile organic compound) profile.

### Reproductive system

Treatments to investigate the reproductive system of *Galeandra blanchetii* were conducted on 10 adult plants during the 2021 and 2022 flowering seasons. Controlled reproductive treatments were performed exclusively in the forest fragment area of the Federal University of Maranhão. The following treatments were applied to intact, bagged flowers: (1) emasculation, in which pollinaria were removed and the flowers were subsequently covered with micromesh bags to test for fruit formation through agamospermy (n = 30); (2) manual cross‐pollination, in which flowers were pollinated using pollinaria from different individuals (n = 30); (3) manual self‐pollination, in which flowers were pollinated using pollinaria from the same flower (n = 30); and (4) spontaneous self‐pollination, in which intact flowers were maintained inside micromesh bags (n = 30). A complete pollinarium was used in each manual pollination event. In total, 120 flowers from 10 plants, distributed among 30 inflorescences, were used in the controlled reproductive treatments.

Fruiting under natural conditions (control treatment), in which flowers remained exposed to natural pollinators, was evaluated in both study areas. In 2021, 42 plants, 10 inflorescences and 20 individuals were monitored, while in 2022, 48 flowers, 10 inflorescences and 20 individuals were evaluated.

Fruit obtained from the different treatments (manual self‐pollination, manual cross‐pollination and natural pollination) were collected to evaluate the viability of formed seeds. For this, seeds were placed in a 1% solution of 2,3,5‐triphenyl tetrazolium in 40‐ml glass vials. After that, the vial was closed and covered with aluminium foil to avoid light interference and incubated in the dark for 24 h in a water bath set to 38 °C (Vudala & Ribas 2017). After incubation, the tetrazolium solution was discarded and the seeds were washed three times with distilled and deionized water for 3 min (Magrini *et al*. 2019). For each fruit obtained from the different pollination treatments, 200 seeds were evaluated under a stereomicroscope to determine seed viability. The relative viability (percentage) was estimated by dividing the number of pinkish/reddish seeds (viable) by the total number of embryos counted. Differences in seed viability among treatments (self‐pollination, cross‐pollination and natural pollination) were analysed using Kruskal–Wallis tests for non‐normally distributed data, followed by Dunn's post hoc tests with Benjamini–Hochberg *P*‐value adjustment (α = 0.05). A boxplot was generated using the R package *ggplot2* (Wickham 2016) to illustrate the variation in seed viability among treatments, consistent with the statistical analyses performed.

## RESULTS

### Morphology and histochemical tests

The flowers are up to 6.5 ± 0.50 cm long; the sepals are free. The labellum is internally covered with unicellular trichomes and forms a prominent spur. The labellum disc has two longitudinal white keels, which direct floral visitors towards the spur. The spur is elongated and ascending and bears trichomes that are longer than those on the labellum margin. The column is club‐shaped, with translucent trichomes near the stigma and two rounded vinaceous projections at the base. The two pollinia are rounded, yellow and attached to an adhesive, falciform disc (Table S2).

Transverse sections of the labellum showed an adaxial surface with a uniseriate epidermis and a thin cuticle. Epidermal cells were cuboidal and bore unicellular trichomes arranged in longitudinal lines from the margin to the spur. This region also exhibited longer unicellular trichomes (Fig. 2B,C). The parenchyma consisted of anisodiametric cells with few intercellular spaces, and vascular bundles were collateral. The abaxial surface was composed of flattened, glabrous cuboidal cells.

**Fig. 2:**
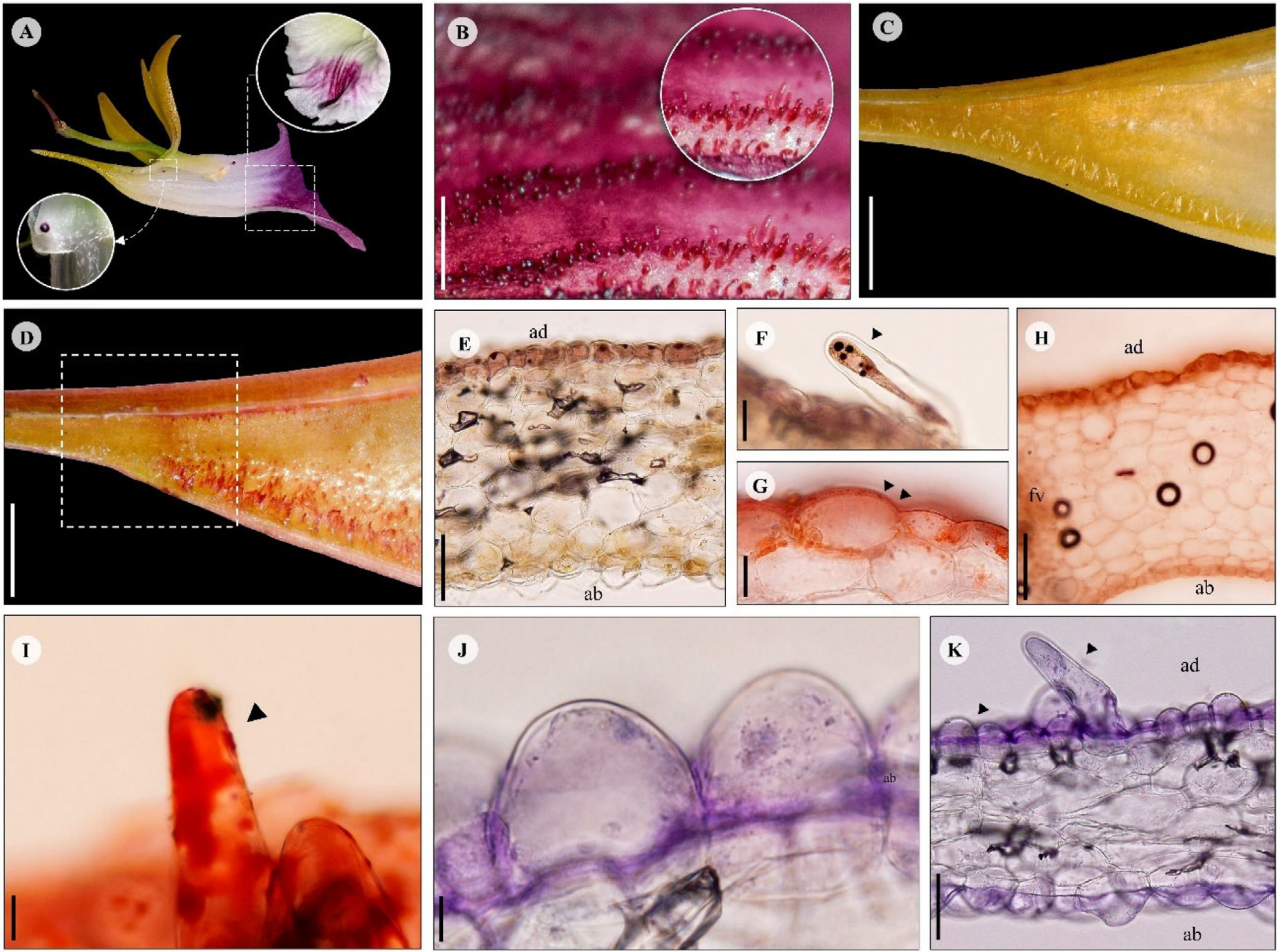
Longitudinal section of the flower of *Galeandra blanchetii* (Orchidaceae). (without staining) (A); Positive tests with neutral red in the labellum, highlighting labellum trichomes (osmophores). Detail of the spur with trichomes with translucent and yellowish coloration (without staining) (C). After staining, the trichomes that were not stained were the ones in the spur (D). Manual sections of the labellum of *G. blanchetii*: uniseriate epidermis with positive Lugol indicating that starch is present only in the adaxial epidermis surface (E) and some grains in the trichomes (F). Positive tests of Sudan IV showing oil droplets in the cuboid epidermal cells (G), with non‐reagent cuboid and isodiametrical parenchyma cells (H) with unicellular trichomes with lipophilic content (I). NADI reagent tested positive for the adaxial epidermis (J) and unicellular trichomes highlighting the presence of terpenes in the internal content (K). Scale bars: (A) = 4 cm; (B–D) = 1 cm; (E, H, K) = 100 μm; (F, G, I, J) = 20 μm.

Neutral red staining indicated high metabolic activity in the labellum, particularly in the adaxial epidermis and unicellular trichomes, except those in the spur (Fig. 2D). Lugol's solution revealed starch grains in adaxial epidermal cells (Fig. 2E), in trichomes (Fig. 2F) and near vascular bundles, but not in the surrounding parenchyma. Proteins, phenolic compounds and mucilage were not detected.

Sudan IV staining highlighted cuticular lipids in the labellum, particularly in adaxial epidermal cells and trichomes (Fig. 2G–I), with no lipophilic compounds detected in the subepidermal parenchyma. NADI reagent revealed terpene‐associated secretion in adaxial epidermal cells containing lipid droplets (Fig. 2J) and in trichomes at the labellum margin and tube (Fig. 2K). The periodic acid–Schiff (PAS) test was negative for polysaccharides in the spur and other regions of the labellum (see Table S3).

### Floral visitors and pollination mechanism

Of the 48 floral visitors recorded, 87.5% were bees (Apidae, n = 42), while 12.5% comprised butterflies (Hesperiidae, n = 2) and ants (n = 4). Among bees, Euglossini was the most frequent tribe, representing 80.9% of bee visitors (n = 34), mainly composed of *Euglossa* (n = 33) and a single individual of *Eulaema*. The remaining bee visitors were Meliponini (n = 5), Ceratinini (n = 2) and Centridini (n = 1) (Table 1; Fig. 3). Most visits occurred between 09:00 and 11:00 (Fig. S4). No pollinarium removal was observed in flowers monitored overnight (17:00–07:00).

**Table 1:** Visitors and pollinators recorded in *Galeandra blanchetii* (Orchidaceae) in natural populations in São Luís, Maranhão, Brazil, between 2018 and 2023.

family/tribe/species	number of visits	behaviour
Apidae (CERATININI)		
*Ceratina* sp	2	EN
*Epicharis* sp	1	EN^a^
Apidae (EUGLOSSINI)		
*Euglossa* sp (♀)	11	EN/CE
*Euglossa amazonica* (Dressler, 1982) (♂)	1	EN
*Euglossa cordata* (Linnaeus, 1758) (♂)	3	EN/SC^a^
*Euglossa securigera* (Dressler, 1982) (♂)	6	EN/SC^a^
*Euglossa* sp (sex indet.)	11	EN
*Eulaema nigrita* (Lepeletier, 1841) (♂)	1	EN
Apidae (MELIPONINI)		
*Trigona fulviventris* (Guérin‐Méneville, 1844) (♀)	5	EN/SE
Hesperiidae		
*Saliana saladin catha* (Evans, 1955)	1	EN
Non‐identified 1	1	EN

Visiting insect behaviour: entered the flower searching for nectar (EN); collection of floral scents (SC); external access to the floral spur (SE).

^a^ Individuals observed removing pollinaria.

**Fig. 3:**
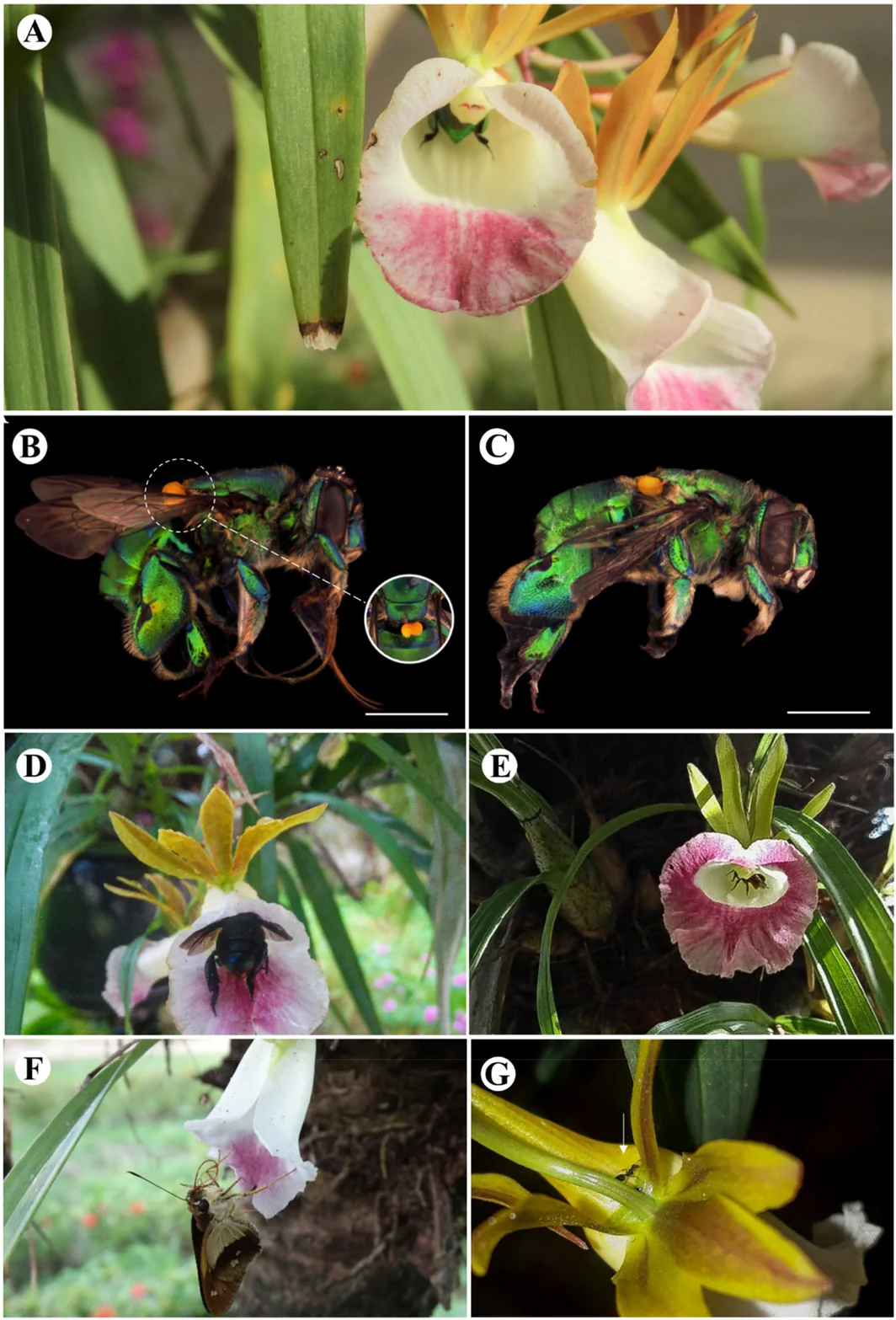
*Euglossa* bee visiting flower of *Galeandra blanchetiii* (Orchidaceae) searching for nectar. Note the alignment between the bee dorsal area and the column (A). *Euglossa* males captured with *G. blanchetii* pollinarium: *Euglossa cordata*, showing the pollinarium in the apical portion of the scutellum (B). *Euglossa securigera* with pollinarium adhered in the apical portion of the scutellum (C). *Eulaema nigrita* bee searching for nectar in *Galeandra blanchetii* (Orchidaceae) flower (D); *Trigona fulviventris* bee exiting the tubular flower (E); Lepidoptera *Saliana saladin catha* visiting (F); *Crematogaster* ant in the sepal‐petal insertion (G).

Within *Euglossa*, 33 individuals were recorded. Most individuals exhibited nectar‐searching behaviour, characterized by an extended proboscis during flower entry and exit and direct movement into the floral tube towards the spur (Video S1). Females generally performed short visits consistent with nectar foraging, whereas males exhibited labellum‐scraping behaviour associated with floral scent collection (Video S2).

Pollinaria were removed only during flower exit, when bees moved backward through the floral tube, allowing the viscidium to attach to the scutellum. Of the 33 *Euglossa* individuals, 22 (10 males and 12 females) fully entered the labellum while searching for nectar (Fig. 3A). One male *Euglossa securigera* removed a pollinarium, and one male *E. cordata* arrived with pollinia attached to the scutellum (Fig. 3B,C). *Euglossa amazonica* exhibited only nectar‐searching behaviour.

Nectar‐searching duration was 0.11 ± 0.06 s (n = 30), whereas scent collection lasted 0.57 ± 0.51 s (n = 3). Meliponini bees (n = 5) exhibited probing behaviour, contacting the labellum, sepals and petals with proboscis and antennae. In some visits, they moved towards the spur and punctured the labellum (Video S3). Only one visit by *Epicharis* sp. resulted in pollinarium removal (Video S4). No pollinarium removal was observed for *Eulaema nigrita*, Meliponini or Ceratinini bees (Fig. 3D,E). The scent‐collecting behaviour observed in males corresponds to the collection of floral perfumes.

### Mechanical fit between bees and flowers

The morphometric analysis of the flowers showed that all bee species, except *Eulaema nigrita* (Lepeletier, 1841), had a narrower mesosoma (2.06–5.03 mm) than the distance between the adaxial portion of the labellum tube and the column stigma (7.4 ± 1.40 mm) (Table S5). However, only *Euglossa amazonica*, *E*. *cordata, E. securigera and Euglossa sp. 1* exhibited mesosoma height (± SD; Table S6) corresponding to the anther region (An) and to the internal floral tube at the labellum floor and paired keels below the rostellum cup (Cn) (Fig. 4).

**Fig. 4:**
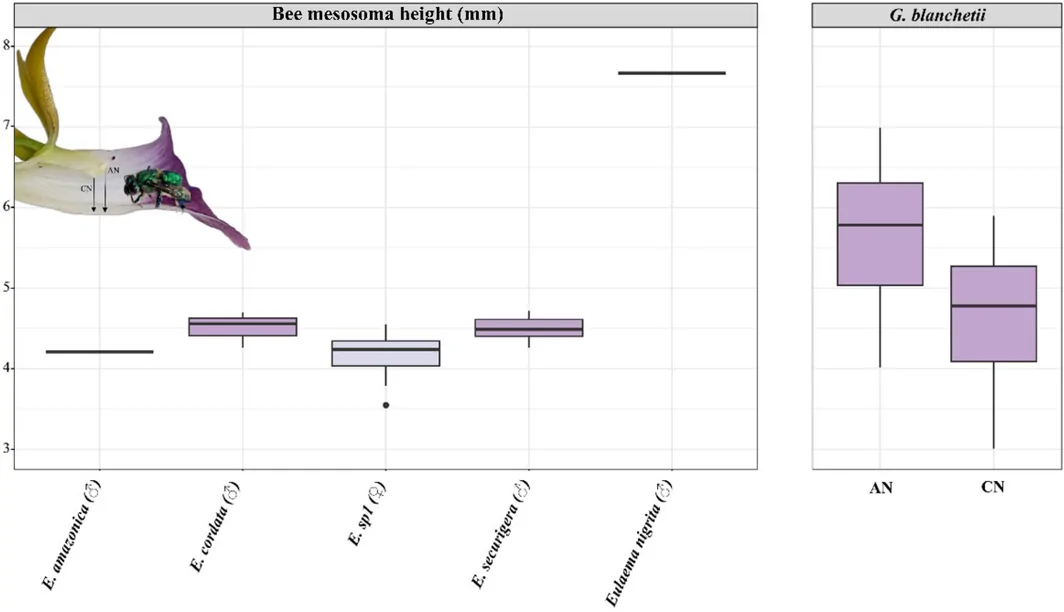
Comparison of the mesosoma heights (mm) of the captured *Euglossa* species with the floral traits ‘AN’ (mm) and ‘CN’ (mm) of *Galeandra blanchetii*. AN represents the labellar tube entrance at the anther region, whereas CN represents the labellar tube width at the callus region below the rostellar flap. Potential pollinators are *Euglossa* species with a mesosoma height that fits within the AN–CN interval and a mesosoma width smaller than the labellar tube width. Effective pollinators are highlighted in purple. The photograph of *Euglossa securigera* illustrates its body position during floral visitation and the region of the mesosoma that contacts the column of *G. blanchetii*. Scale bars (B–C): 5 mm.

All male and female *Euglossa* bees had a glossa length (Table S6) equal to or shorter than the spur length (12.9 ± 1.77 mm) of the flowers (Table S5). Therefore, individuals of *Euglossa* were required to enter the labellum to access the spur.

In contrast, male *Eulaema nigrita* exhibited a long proboscis (11.23 mm) and a thorax height (7.6 mm) that exceeded the distance between the column stigma and the adaxial surface of the labellum tube (4.6 mm). Together with the observed visitation behaviour, these traits indicate that individuals did not require full entry into the floral tube to access the spur. Visits lasted approximately 1 s, and the species was recorded only three times during the study period, always in the morning. Under these conditions, pollinarium removal was not observed.

### Chemical composition of the floral perfume

The GC–MS analyses revealed a total of 34 compounds in the floral scent bouquet of *G. blanchettii*, of which 17 were identified based on comparison with authentic standards, 12 were tentatively identified, and five remained unidentified (Table 2). Benzenoids were the most representative compound class (12 compounds), followed by sesquiterpenes/sesquiterpenoids (10), aliphatic compounds (4), monoterpenes (2), norisoprenoids (1) and unidentified compounds (5) (Table 2). Methyl salicylate and methyl benzoate were the predominant constituents of the floral bouquet, representing 55.0 ± 12.91% (N = 8) and 28.2 ± 22.50% (N = 8) of the total volatile emission, respectively.

**Table 2:** Mean relative amount of floral scent constituents of *Galeandra blanchetii* identified by GC–MS.

compound		RI (calc)	RI (lit)	mean (%) ± SD
Aliphatics				
1	6‐methyl‐5‐hepten‐2‐one*	988	987	1.61 ± 4.56
2	Nonanal*	1,105	1,107	0.64 ± 1.32
3	Decanal*	1,206	1,208	0.26 ± 0.74
4	Hexadecanal*	1,817	1,818	0.18 ± 0.52
Monoterpenes				
5	Limonene*	1,027	1,030	0.08 ± 0.23
6	Bergamal	1,058	1,053	0.31 ± 0.89
Aromatic compounds				
7	Benzaldehyde*	959	960	2.73 ± 2.57
8	Benzyl alcohol*	1,035	1,040	0.26 ± 0.52
9	Methyl benzoate*	1,094	1,096	28.2 ± 22.50
10	Phenethyl alcohol*	1,113	1,113	0.21 ± 0.45
11	Benzyl acetate*	1,166	1,167	0.11 ± 0.24
12	Ethyl benzoate*	1,171	1,170	0.78 ± 1.29
13	Methyl salicylate*	1,195	1,192	55.0 ± 12.91
14	2‐Phenylethyl acetate*	1,259	1,257	0.67 ± 1.90
15	4‐Methoxyphenethyl acetate	1,511	1,517	1.37 ± 3.87
16	(3Z)‐hexenyl benzoate	1,573	1,573	0.10 ± 0.28
17	Hexyl benzoate	1,580	1,581	0.25 ± 0.70
18	Benzyl benzoate*	1,767	1,772	0.46 ± 1.30
Sesquiterpenes and sesquiterpenoids				
19	δ‐Elemene	1,335	1,337	0.02 ± 0.07
20	β‐Bourbonene	1,385	1,382	0.13 ± 0.37
21	β‐Elemene	1,393	1,390	0.16 ± 0.45
22	β‐Copaene*	1,430	1,433	0.12 ± 0.33
23	cis‐Muurola‐4(14),5‐diene	1,464	1,466	0.06 ± 0.18
24	γ‐Amorphene	1,482	1,490	0.61 ± 1.26
25	Putative ε‐amorphene	1,498	1,502	0.16 ± 0.44
26	α‐Farnesene	1,509	1,504	0.47 ± 1.04
27	(E)‐Nerolidol*	1,565	1,561	0.30 ± 0.84
28	Methyl farnesoate	1,788	1,780	2.17 ± 5.88
Norisoprenoids				
29	Geranylacetone	1,454	1,456	0.74 ± 1.16
Unidentified compounds				
30	NI (m/z 41,81,93,147,189,204)	1,382	‐	0.07 ± 0.21
31	NI (m/z 41,105,120,133,161,204)	1,420	‐	0.40 ± 0.74
32	NI (m/z 41,105,161,189,204)	1,445	‐	0.16 ± 0.44
33	NI (m/z 41,105,119,134,159,204)	1,524	‐	1.10 ± 2.94
34	NI (m/z 41,55,69,98,166,248)	1,996	‐	0.20 ± 0.57

RI (calc) calculated retention indexes, RI (lit) retention indices taken from literature, NI: compound not identified. Asterisk (*) refers to compounds identified based on comparison with authentic standards.

### Reproductive system

*Galeandra blanchetii* is self‐compatible, but no fruit set was observed following spontaneous self‐pollination or emasculation (agamospermy) treatments. Natural fruit set was 19% in 2021 (n = 42 flowers) and 17% in 2022 (n = 46 flowers) (Table S7). Manual self‐pollination and manual cross‐pollination resulted in 100% fruit set, and at least 60% of the seeds were viable in both treatments, as determined by the proportion of seeds with stained embryos. Most seeds exhibited well‐developed embryos; however, seed viability differed significantly among the manual self‐pollination, manual cross‐pollination and natural pollination (control) treatments (X^2^_kw_ = 49.815, df = 2, *P* < 0.05). A significantly higher proportion of seeds from cross‐pollinated and naturally pollinated flowers (control) exhibited viable embryos compared with those from self‐pollinated flowers, which had a higher proportion of seeds lacking embryos or containing aborted embryos (Benjamini–Hochberg‐corrected Dunn's test, *P* < 0.05) (Fig. 5). No significant difference was detected between manual cross‐pollination and natural pollination (control) (Dunn's test, *P* = 1.00).

**Fig. 5:**
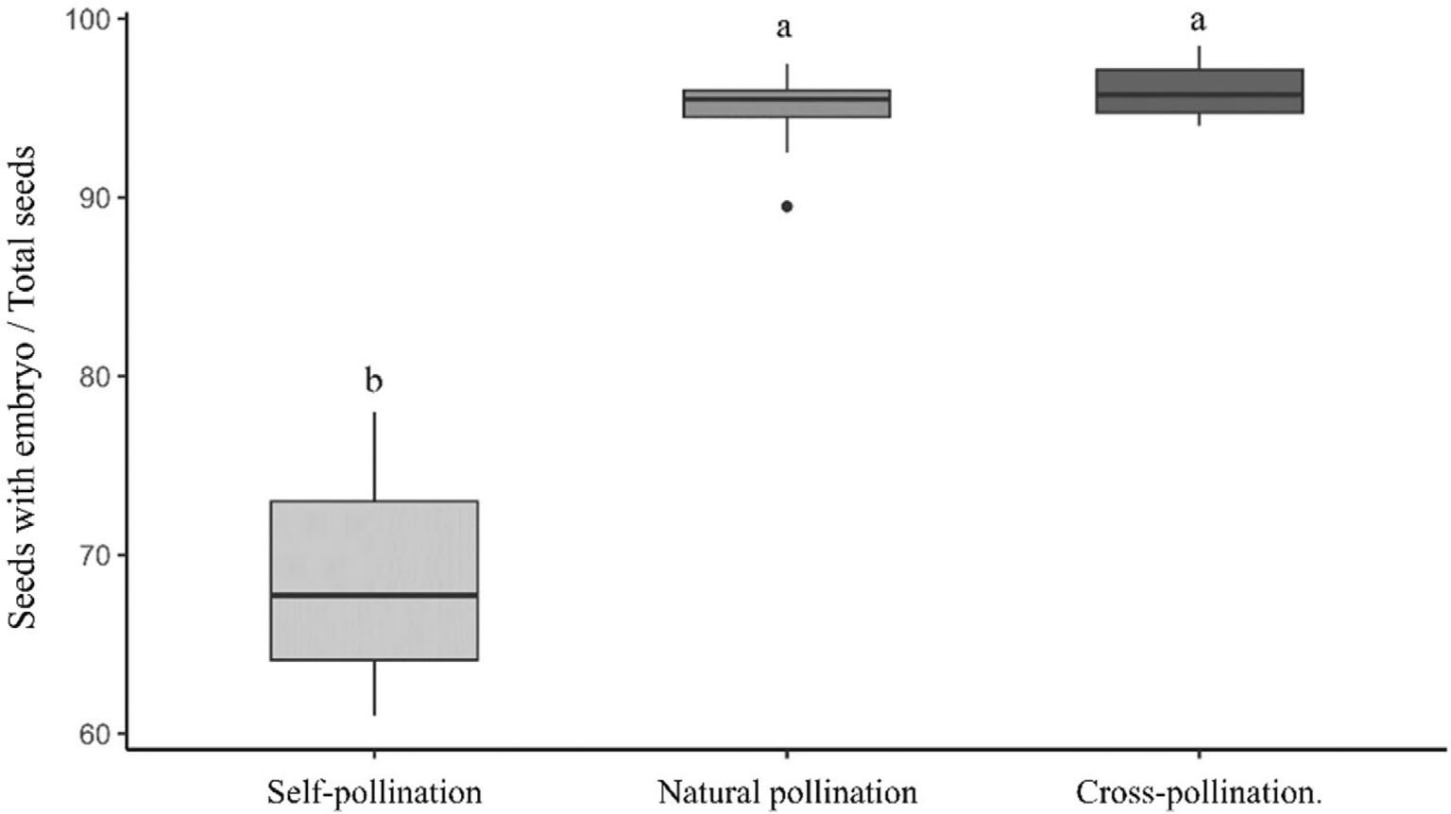
Seed viability of *Galeandra blanchetii* (Orchidaceae). The x‐axis shows the treatments: self‐pollination (n = 20), natural pollination (n = 20) and cross‐pollination (n = 20). The y‐axis shows the percentage of seeds containing embryos. Medians are represented by central horizontal lines; boxes indicate the first and third quartiles, and whiskers represent the range. Different letters indicate significant differences according to Dunn's test (*P* < 0.05).

## DISCUSSION

Despite recent advances in understanding pollination in *Galeandra*, including evidence of floral mimicry in *G. montana* (Cardoso *et al*. 2026), the floral scent chemistry of the genus remains poorly characterized. Here, we provide the first chemical characterization of floral scent in *Galeandra* and integrate these data with floral morphology, floral biology and pollinator effectiveness to investigate the pollination system of *G. blanchetii*.

Our results support our initial hypothesis and demonstrate that the pollination system of *G. blanchetii* shares characteristics with other Catasetinae species that offer floral fragrances as rewards while also exhibiting uncommon melittophilous traits. The combination of melittophilous floral features and the absence of nectar suggests that *G. blanchetii* relies on mechanical fit together with visual and olfactory cues to deceive floral visitors. At the same time, the production of floral perfumes indicates affinity with perfume‐mediated pollination systems. The behavioural responses of fragrance‐collecting bees, as well as their precise interactions with the flowers, support their role as effective pollinators. Considering the combination of deceptive floral traits, perfume‐related cues and the specific guild of floral visitors associated with *G. blanchetii*, our findings provide new insights into how deceptive and perfume‐based pollination strategies may have diversified and become integrated during the evolutionary history of *Galeandra*.

### Deceptive traits and perfume cues shaping the pollination strategy of *G. blanchetii*

*Galeandra* species are generally considered part of the group of orchids pollinated through food deception (Ackerman 1986; Jersáková *et al*. 2006; Pansarin & Maciel 2017; Cardoso *et al*. 2026), as their flowers possess a spur without visible nectar. A recent study of *Galeandra montana* based on anatomical and histochemical analyses supported the absence of nutritional floral rewards, while providing evidence that floral deception in this species involves Batesian floral mimicry of *Jacaranda rufa* (Bignoniaceae) (Cardoso *et al*. 2026). These findings reinforce the association between nectar absence and deceptive pollination in the *Galeandra* species studied, while also indicating that different deceptive strategies may occur within the genus. Our results on the floral biology of *G. blanchetii* extend this interpretation beyond food deception, as its flowers also produce floral scents that may serve as resources for male Euglossini bees.

In orchids, osmophores are typically distributed on the adaxial surfaces of the sepals, petals, labellum or column (Bernhardt & Burns‐Balogh 1986; Vogel 1990; Dressler 1993). In *G. blanchetii*, the labellum was the floral structure with the strongest scent emission and contained epidermal osmophores and unicellular glandular trichomes, both of which exhibited the accumulation of lipidic and terpenoid substances associated with floral scent production (Williams & Whitten 1983). In addition, the longitudinal arrangement of trichomes may function as a resource guide, as reported for species of *Vanilla* (Pansarin 2022; Pansarin & Ferreira 2022), potentially directing floral visitors towards the spur region. A similar arrangement of trichomes has been reported in *G. montana*, where the median region of the adaxial labellum surface bears parallel, raised ridges extending towards the column (Cardoso *et al*. 2026), suggesting that this floral trait may be recurrent within *Galeandra*.

However, the floral parenchyma of *G. blanchetii* was not secretory, differing from the condition described in species of *Catasetum* (Franken *et al*. 2016; Reposi *et al*. 2021) and *Stanhopea* (Anton *et al*. 2012), but agreeing with the condition reported for *G. montana* (Cardoso *et al*. 2026). Volatile release in *G. blanchetii* appeared to occur through a thin cuticle, as no stomata or pores were observed, a mechanism commonly reported in orchid species pollinated by male Euglossini bees (Dudareva & Pichersky 2006; Anton *et al*. 2012; Pansarin *et al*. 2014). Starch grains were observed only in the secretory epidermis and in regions adjacent to the vascular bundles, but not in the surrounding parenchyma. The presence of starch associated with osmophores has also been reported in *Stanhopea*, *Cycnoches* and *Vanilla* (Anton *et al*. 2012; Pansarin 2022), where it has been interpreted as an energy source for volatile synthesis (Vogel 1963; Stern *et al*. 1987; Dudareva & Pichersky 2006).

Taken together, these findings suggest that the combination of deceptive visual traits and perfume‐related cues underlies a pollination strategy that integrates food deception with the use of floral fragrances as resources. This mixed strategy may reflect selective pressures similar to those acting in other Catasetinae lineages, suggesting that *G. blanchetii* occupies an informative position along the continuum between purely deceptive and perfume‐mediated pollination systems. Whether this condition represents a transitional stage in the evolution of perfume‐mediated pollination or an alternative strategy that enhances floral visitation and reproductive success remains an open question requiring further comparative studies within the genus.

### Floral scent and pollinator‐mediated reproduction

Floral scent is an important attractant in pollination systems involving male Euglossini bees (Dressler 1982; Sazima *et al*. 1993). A diverse range of volatile compounds can mediate such interactions (Dressler 1982; Nunes *et al*. 2016; Brandt *et al*. 2020; Opedal *et al*. 2020). The major constituents of the floral scent of *G. blanchetii* are methyl salicylate and methyl benzoate, both considered attractants in several orchid species pollinated by male Euglossini bees (*e.g*., Hills *et al*. 1972; Gerlach & Schill 1991; Hetherington‐Rauth & Ramírez 2016; Nunes *et al*. 2016; Milet‐Pinheiro *et al*. 2018, 2022), including records of methyl salicylate baits attracting *Euglossa securigera* (Dressler 1982) (Martins *et al*. 2018) and *Eulaema nigrita* (Lepeletier 1841) (Brandt *et al*. 2021) and of methyl benzoate being a strong attractant for *Euglossa cordata* (Linnaeus, 1758) (Brandt *et al*. 2021).

Nonanal was also detected in *G. blanchetii* in low relative abundance and has been associated with floral signals related to nectar rewards in some angiosperms (Liu *et al*. 2022). However, given its widespread occurrence in plant volatile blends, its presence alone does not provide strong evidence for a role in deceptive signalling in *G. blanchetii*. Comparative data from other *Galeandra* species are currently limited. In *Galeandra minax* Rchb.f., Gerlach & Schill (1991) identified benzyl acetate and 1,8‐cineole as floral volatiles. In *G. blanchetii*, benzyl acetate was the only compound shared with *G. minax*, whereas the remaining compounds differed between the two species. This compositional divergence suggests interspecific variation in floral scent within *Galeandra* and highlights the need for broader comparative studies to assess its potential taxonomic and evolutionary significance.

The use of floral scents as a pollination resource in *G. blanchetii* was confirmed by the observation of a male *Euglossa securigera* collecting volatile oils on the surface of the labellum (Video S2). The scraping process to remove scent is performed by male Euglossini bees with their forelegs (Dodson 1962; Dressler 1982); the scent is then transferred to the middle legs and from those to the dilated tibia of the hind legs (or pollen basket) (Vogel 1963; Landim *et al*. 1965; Eltz *et al*. 1999). These scents are used by male bees during mating rituals, increasing the chances of mating and generating more descendants (Henske *et al*. 2023). However, these bee species do not depend exclusively on orchids to acquire scents, because they can collect them from other flowering plants or from extrafloral sources, such as fruits, mushrooms and decaying wood (Whitten *et al*. 1993; Pemberton & Wheeler 2006; Cappellari & Harter‐Marques 2010; Karremans *et al*. 2023).

From a reproductive plant perspective, bees enter the spur in search of nectar and remove the pollinarium upon exiting the flower, where it adheres to the scutellum. In this study, both males and females were observed entering the labellum with an extended glossa while searching for nectar, supporting a food‐deception mechanism in *Galeandra*. Only males of two Euglossa species (*E. securigera* and *E. cordata*) effected pollinarium transfer, consistent with previous records of *Galeandra* pollinaria on Euglossa bees (Pearson & Dressler 1985; Ramírez *et al*. 2011), indicating that some species in this genus act as effective pollinators.

Pollination by male Euglossa bees is relatively specific, being mainly driven by chemical cues and, secondarily, by morphological compatibility and behavioural traits that enable pollinarium transport during visits (Nunes *et al*. 2017; Watteyn *et al*. 2022, 2023; Ackerman *et al*. 2023). Despite their frequent visits, no pollinarium transport was observed in females, likely because their smaller thorax height (4.1 ± 0.29 mm) compared with that of males (Table S5) may hinder effective pollinarium removal. Observations in *G. montana* further suggest that the interaction between floral architecture and bee body size may be important for pollinarium attachment in *Galeandra*. During visits, the unfused portion of the labellum, positioned anterior to the column insertion point, undergoes a slight displacement that accommodates the bee's body size. This adjustment establishes a close mechanical fit between the flower and the visitor, so that, as the bee leaves the flower, the viscidium contacts the dorsal surface of the thorax and facilitates pollinarium attachment (Cardoso *et al*. 2026).

Trigona bees mainly probed the labellum trichomes, exhibiting foraging behaviour (Video S5); however, *G. blanchetii* lacks food‐rewarding trichomes (Krahl *et al*. 2019). Nectar‐searching behaviour was more frequent than scent‐collecting behaviour, contrasting with Vanilla species, which provide both nectar and floral fragrance and thus elicit both behaviours, whereas *Galeandra* offers floral fragrance only.

The controlled pollination experiments demonstrated that *G. blanchetii* is a self‐compatible species. However, the absence of fruit formation in both agamospermy and spontaneous self‐pollination treatments, contrasted with successful fruit set under biotic conditions, indicates a strong dependence on pollinators for reproductive success. This pattern is consistent with the low natural fruit set observed in many tropical orchids (Krahl *et al*. 2019; Albuquerque *et al*. 2021; Reyes *et al*. 2021) and suggests that the reproductive output in *G. blanchetii* is primarily pollinator‐limited.

Although fruit set in *G. blanchetii* was higher than that reported for several orchids pollinated via deceptive mechanisms (Pellegrino *et al*. 2005; Pansarin *et al*. 2008; Suetsugu *et al*. 2015; Caballero‐Villalobos *et al*. 2017; Aguiar & Pansarin 2019), as well as in some fragrance‐rewarding genera such as *Dichaea* (Nunes *et al*. 2016), *Catasetum* (Milet‐Pinheiro *et al*. 2015; Brandt *et al*. 2020), *Gongora* (Martini *et al*. 2003) and *Zygopetalum* (Nunes *et al*. 2017), these differences likely reflect variation in pollinator availability and interaction specificity among systems rather than reproductive autonomy. In addition, these reproductive patterns may be influenced by floral signalling strategies, where nectar‐searching and fragrance‐collecting behaviours contribute to increased visitation rates. A similar dual‐attraction system has been reported in other orchid lineages with mixed reward strategies (*e.g*., Martel *et al*. 2019; Milet‐Pinheiro *et al*. 2021; Pontes *et al*. 2022), where multiple sensory cues enhance pollinator attraction.

### *Galeandra blanchetii* as an intermediate step in the evolution of perfume‐mediated pollination

Phylogenetically, *Galeandra* was resolved within Catasetinae as sister to *Cyanaeorchis* and *Grobya*, both of which are associated with nutritive floral resources (Pansarin & Maciel 2017). In contrast, *G. blanchetii* exhibited a combination of deceptive floral traits and the production of complex perfume blends. This phylogenetic context highlights the diversity of pollination strategies within this lineage and provides a broader framework for interpreting the coexistence of deceptive and olfactory cues in *G. blanchetii*. A comparable system has been reported in *Vanilla*, in which pollinator attraction integrates floral fragrance rewards and food deception (Watteyn *et al*. 2022), supporting the possibility that such mixed mechanisms may be more widespread across orchid lineages than previously assumed.

Species within *Galeandra* comprise both terrestrial and epiphytic taxa and exhibit marked ecological and morphological variation, including substantial differences in floral spur length. Comparative analyses indicate that spur length is not a conserved character in *Galeandra* but has undergone repeated modifications across lineages, potentially reflecting shifts in ecological conditions and pollinator‐mediated selective pressures (Martins *et al*. 2018). Such variation may also be consistent with differences in nectar production, as suggested by phylogenetic reconstructions in other orchid genera such as *Dendrobium* (Jia & Huang 2022).

Recent findings from the terrestrial *Galeandra montana* provide an additional perspective on the relationship between spur morphology, reward availability and pollinator identity. In this species, the spur is short and obtuse; no nectar was detected, and pollination occurs through food deception mediated by Batesian floral mimicry (Cardoso *et al*. 2026). Its pollinators, including *Xylocopa* and *Bombus*, have shorter proboscides than Euglossini bees. In contrast, *G. blanchetii* has a relatively long spur and was effectively pollinated by male Euglossa species, which have longer proboscides. These observations raise the possibility that spur length in *Galeandra* may be associated with pollinator identity and pollination strategy, with longer spurs potentially being associated with interactions with Euglossini bees, whereas shorter spurs may occur in species pollinated by bees with comparatively shorter proboscides and relying on food deception. Further comparative studies across the genus are needed to test this association and clarify the relationships among spur morphology, pollinator identity and pollination strategy.

Collectively, these findings indicate that *G. blanchetii* represents a putative transitional condition in the evolution of perfume‐mediated pollination, in which deceptive and reward‐associated olfactory cues co‐occur and may jointly enhance attraction of fragrance‐sensitive bees. The behavioural responses of its pollinators, together with the species' strong reproductive dependence on these bees, further suggest that selection favouring perfume collection was likely already acting within this lineage. Overall, *Galeandra* emerges as a valuable system for understanding how floral signals and pollinator interactions have been assembled and modified through evolutionary time, particularly in association with ecological shifts and spur‐related morphology.

## CONCLUSIONS

Despite being self‐compatible, *Galeandra blanchetii* relies exclusively on biotic vectors for reproduction. Our results revealed a dual‐attraction system involving floral volatiles and food deception, with both male and female Euglossini attracted to the flowers. Effective pollination, however, was restricted to males of *Euglossa securigera*, *E. cordata* and one Centridini species (*Epicharis* sp.). Although males collected floral volatiles from the labellar osmophores, pollination occurred only when they subsequently entered the non‐functional spur while searching for nectar, resulting in pollinarium removal and deposition during floral exit. This represents the first record of male *Euglossa*‐mediated pollination in *Galeandra*.

Reproductive success in *G*. *blanchetii* depends on the coordinated action of olfactory attraction and floral traits associated with food deception, rather than on volatile collection alone. While scent‐mediated signalling aligns with patterns observed in Catasetinae, the presence of melittophilous traits uncommon in the group indicates a more complex integration of attraction pathways. Comparative studies across *Galeandra*, considering variation in habitat, pollinator assemblages and spur length between terrestrial and epiphytic species, will be important for assessing the prevalence and evolutionary significance of this dual system and its role in floral diversification.

## CONFLICT OF INTEREST

The authors declare that they have no known competing financial interests or personal relationships that could have appeared to influence the work reported in this paper.

## AUTHOR CONTRIBUTIONS

DMC: Conceptualization; DMC, EMP, ICM: Methodology; DMC, AWCF: Investigation; DMC, EMP: Resources; DMC, EMP, AWCF: Data validation; AD‐M, PM‐P: Support in chemical analyses; APSC: Support in histochemical analyses; DMC: Writing – original draft; DMC, AWCF, APSC, AD‐M, SA‐L, PM‐P, ICM, EMP: Writing – review and editing; DMC, SA‐L: Visualization, including review of images and videos. All authors approved the final version of the manuscript.

## Supporting information


**Fig. S1.** Map of the state of Maranhão highlighting the municipality of São Luís, where the study was conducted. (A) shows the study site at the Federal University of Maranhão, and (B) shows the study site at the Environmental Protection Area of Itapiracó, both of which were used for field observations of *Galeandra blanchetii* (Orchidaceae).

**Fig. S2.** Climatic normal for precipitation and temperature in São Luís, MA from 1991 to 2020 (Conventional station, COD: 82280, INMET).

**Fig. S3.** Whole flower of *Galeandra blanchetii* (Orchidaceae) (A); longitudinal section showing the measured floral traits: SL (spur length), LH (labellum height), LRo (distance between labellum and rostellum) and LAn (distance between labellum and anther).

**Fig. S4.** Total visitation frequency and period of visitation in flowers of *Galeandra blanchetii* (Orchidaceae) (flowering periods from 2018 to 2023) in Amazon Forest fragments, northeastern Brazil.

**Table S1.** Summary of observations of *Galeandra blanchetii* (Orchidaceae) carried out on the field in two study areas in São Luís, Maranhão, Brazil. *UFMA: Universidade Federal do Maranhão; APA Itapiracó: Environmental Protection Area Itapiracó.
**Table S2**. Morphological measures of *Galeandra blanchetii* (Orchidaceae) in populations in São Luís, Maranhão, Brazil.
**Table S3**. Histochemical analysis of flowers of *Galeandra blanchetii* (Orchidaceae).
**Table S4**. Percentage of *Euglossa* bees observed (including not collected) in relation to the frequency of search for nectar or scent in *Galeandra blanchetii* (Orchidaceae) in São Luís, Maranhão, Brazil.
**Table S5**. Measures of *Galeandra blanchetii* (Orchidaceae) flowers (N = 30). AN: Height from the labellum up to the mean anther region; CN: Height from the labellum to the rostellum cup; LT: Floral tube width; and CC: Spur lenght.
**Table S6**. Measurements performed on bees collected in *Galeandra blanchetii* (Orchidaceae) flowers, in natural populations in Maranhão, Brazil.
**Table S7**. Result of fruit formation in *Galendra blanchetii* (Orchidaceae) according to experimental treatments and natural conditions in plants from two study locations in São Luís, in the state of Maranhão, Brazil.

**Video S1.** Visits of female *Euglossa* searching for nectar in *Galeandra blanchetii* flower.

**Video S2**. Male *Euglossa* collecting scent in *Galeandra blanchetii* flower.

**Video S3**. Male searching for nectar in *Galeandra blanchetii* flower.

**Video S4**. *Epicharis* searching for nectar in *Galeandra blanchetii* flower.

**Video S5**. *Trigona* searching for nectar in *Galeandra blanchetii* flower.

## Data Availability

Data sharing not applicable to this article as no datasets were generated or analysed during the current study.

## References

[plb70288-bib-0001] Ackerman J.D. (1986) Coping with the epiphytic existence: pollination strategies. Selbyana, 9, 52–60.

[plb70288-bib-0002] Ackerman J.D. , Phillips R.D. , Tremblay R.L. , Karremans A. , Reitr N. , Peter C.I. , Bogarín D. , Pérez‐Escobar O.A. , Liu H. (2023) Beyond the various contrivances by which orchids are pollinated: global patterns in orchid pollination biology. Botanical Journal of the Linnean Society, 202, 295–324.

[plb70288-bib-0107] Adams R.P. (2007) Identification of essential oil components by gas chromatography/mass spectrometry, Vol. 456. Allured publishing corporation, Carol Stream, IL, USA, pp 544–545.

[plb70288-bib-0004] Aguiar J.M.R.B.V. , Pansarin E.R. (2019) Deceptive pollination of *Ionopsis utricularioides* (Oncidiinae: Orchidaceae). Flora, 250, 72–78.

[plb70288-bib-0005] Albuquerque N.S.L. , Milet‐Pinheiro P. , Cruz D.D. , Navarro D.M.A.F. , Machado I.C. (2021) Pollination of the strongly scented *Sarcoglottis acaulis* (Orchidaceae) by male orchid bees: nectar as resource instead of perfume. Plant Biology, 23, 719–727. 10.1111/plb.13297 34171178

[plb70288-bib-0006] Anton S. , Kaminska M. , Stpiczynska M. (2012) Comparative structure of the osmophores in the flower of *Stanhopea graveolens* Lindley and *Cycnoches chlorochilon* Klotzsch (Orchidaceae). Acta Agrobotanica, 65, 11–22.

[plb70288-bib-0109] Ayasse M. (2023) Chemical ecology: male orchid bees attract females with environmental‐derived perfumes in courtship display. Current Biology, 33(10), R405–R407. 10.1016/j.cub.2023.04.016 37220732

[plb70288-bib-0110] Batista J.A.N. , Mota A.C.M. , Proite K. , Bianchetti L.D.B. , Romero‐González G.A. , Huerta H. , Salazar G.A. (2014) Molecular phylogenetics of Neotropical *Cyanaeorchis* (Cymbidieae, Epidendroideae, Orchidaceae): geographical rather than morphological similarities plus a new species. Phytotaxa, 156(5). 10.11646/phytotaxa.156.5.1

[plb70288-bib-0112] Bernhardt P. , Burns‐Balogh P. (1986) Observations of the floral biology of *Prasophyllum odoratum* (*Orchidaceae, Spiranthoideae*). Plant Systematics and Evolution, 153(1‐2), 65–76. 10.1007/bf00989418

[plb70288-bib-0111] Bisrat D. , Jung C. (2022) Roles of flower scent in bee? flower mediations: a review. Journal of Ecology and Environment, 46. 10.5141/jee.21.00075

[plb70288-bib-0008] Blaauw B.R. , Isaacs R. (2014) Larger patches of diverse floral resources increase insect pollinator density, diversity, and their pollination of native wildflowers. Basic and Applied Ecology, 15, 701–711.

[plb70288-bib-0010] Brandt K. , Machado I.C. , Navarro D.M.D.A.F. , Dötterl S. , Ayasse M. , Milet‐Pinheiro P. (2020) Sexual dimorphism in floral scents of the neotropical orchid *Catasetum arietinum* and its possible ecological and evolutionary significance. AoB Plants, 12, plaa030.

[plb70288-bib-0113] Brandt K. , Dötterl S. , Ramírez S.R. , Etl F. , Machado I.C. , Navarro D.M.d.A.F. , Dobler D. , Reiser O. , Ayasse M. , Milet‐Pinheiro P. (2021) Unraveling the olfactory biases of male euglossine bees: species‐specific antennal responses and their evolutionary significance for perfume flowers. Frontiers in Ecology and Evolution, 9. 10.3389/fevo.2021.727471

[plb70288-bib-0011] Caballero‐Villalobos L. , Silva‐Arias G.A. , Buzatto C.R. , Nervo M.H. , Singer R.B. (2017) Generalized food‐deceptive pollination in four *cattleya* (Orchidaceae: Laeliinae) species from southern Brazil. Flora, 234, 195–206.

[plb70288-bib-0012] Cappellari S.C. , Harter‐Marques B. (2010) First report of scent collection by male orchid bees (Hymenoptera: Apidae: Euglossini) from terrestrial mushrooms. Journal of the Kansas Entomological Society, 83, 264–266.

[plb70288-bib-0015] Costa L.B.D.S. , Pires C.D.S. , Anjos J.S.D. , Correia B.E.F. , Almeida E.B.D. (2017) Floristic survey of ornamental plants used in Dom Delgado University City at the Universidade federal do Maranhão, São Luís, Maranhão state, Brazil. Ornamental Horticulture, 23, 451–459.

[plb70288-bib-0114] Cardoso J.C.F. , Matallana‐Puerto C.A. , Trevizan R. , Telles F. , Pereira J.P.S. , Oliveira P.E. , Gonçalves R.V. , Duarte M.O. (2026) Impostors in South America: a new Batesian floral mimicry system involving orchids and Bignoniaceae models. Annals of Botany. 10.1093/aob/mcag096 41995601

[plb70288-bib-0016] Costa L.B.S. , Almeida E.B. (2020) Checklist of phanerogamic flora and mapping of areas of urban forest fragments in São Luís, Maranhão. Revista Ecuador, 9, 26–39.

[plb70288-bib-0017] David R. , Carde J.P. (1964) Histochimie‐coloration differentielle des inclusions lipidiques et terpeniques des pseudophylles du pin maritime au moyen du reactif NADI. Comptes Rendus Hebdomadaires Des Seances De L'Academie Des Sciences, 258, 1338.

[plb70288-bib-0018] Davies K.L. , Turner M.P. (2004) Pseudopollen in *Dendrobium unicum* Seidenf. (Orchidaceae): reward or deception? Annals of Botany, 94, 129–132. 10.1093/aob/mch118 PMC4242374 15159216

[plb70288-bib-0019] Davies K.L. , Turner M.P. , Gregg A. (2003) Lipoidal labellar secretions in Maxillaria Ruiz & Pav. (Orchidaceae). Annals of Botany, 91, 439–446. 10.1093/aob/mcg038 PMC4241060 12588723

[plb70288-bib-0108] de Araújo W.S. , Silveira L.T. , de Morais Belchior Couto M. , Falcão L.A.D. , Fagundes M. , de Neves F.S. (2024) Beta diversity of plant–herbivore interactions is unaffected by urbanization levels in Brazilian Cerrado. Arthropod‐Plant Interactions, 18(5), 881–889. 10.1007/s11829-024-10079-0

[plb70288-bib-0020] Dodson C.H. (1962) The importance of pollination in the evolution of the orchids of tropical America. American Orchid Society Bulletin, 31, 525–534.

[plb70288-bib-0021] Dressler R.L. (1982) Biology of the orchid bees (Euglossini). Annual Review of Ecology and Systematics, 13, 373–394.

[plb70288-bib-0022] Dressler R.L. (1993) Phylogeny and classification of the orchid family. Dioscorides Press, Portland, Oregon, USA.

[plb70288-bib-0023] Dudareva N. , Pichersky E. (2006) Floral scent metabolic pathways: their regulation and evolution. In: Dudareva N. , Pichersky E. (Eds), Biology of floral scent. CRC Press, Boca Raton, pp 55–78.

[plb70288-bib-0024] Eltz T. , Whitten W.M. , Roubik D.W. , Linsenmair K.E. (1999) Fragrance collection, storage, and accumulation by individual male orchid bees. Journal of Chemical Ecology, 25, 157–176.

[plb70288-bib-0025] Feder N.E.D. , O'brien T.P. (1968) Plant microtechnique: some principles and new methods. American Journal of Botany, 55, 123–142.

[plb70288-bib-0027] Fisher D.B. (1968) Protein staining of ribboned epon sections for light microscopy. Histochemie, 16, 92–96. 10.1007/BF00306214 4180491

[plb70288-bib-0028] Franken E.P. , Pansarin L.M. , Pansarin E.R. (2016) Osmophore diversity in the *Catasetum cristatum* alliance (Orchidaceae: Catasetinae). Lankesteriana, 16, 317–327.

[plb70288-bib-0030] Gerlach G. , Schill R. (1991) Composition of orchid scents attracting euglossine bees. Botanica Acta, 104, 379–384.

[plb70288-bib-0115] Gomiz N.E. , Torretta J.P. , Aliscioni S.S. (2017) New evidence of floral elaiophores and characterization of the oil flowers in the subtribe Oncidiinae (Orchidaceae). Plant Systematics and Evolution, 303(4), 433–449. 10.1007/s00606-016-1382-7

[plb70288-bib-0031] Gregory M. , Baas P. (1989) A survey of mucilage cells in vegetative organs of the dicotyledons. Israel Journal of Botany, 38, 125–174.

[plb70288-bib-0032] Henske J. , Saleh N.W. , Chouvenc T. , Ramírez S.R. , Eltz T. (2023) Function of environment‐derived male perfumes in orchid bees. Current Biology, 33, 2075–2080. 10.1016/j.cub.2023.03.060 37054713

[plb70288-bib-0033] Hetherington‐Rauth M.C. , Ramírez S.R. (2016) Evolution and diversity of floral scent chemistry in the euglossine bee‐pollinated orchid genus *Gongora* . Annals of Botany, 118, 135–148. 10.1093/aob/mcw072 PMC4934395 27240855

[plb70288-bib-0034] Hills H.G. , Williams N.H. , Dodson C.H. (1972) Floral fragrances and isolating mechanisms in the genus *Catasetum* (Orchidaceae). Biotropica, 4, 61–76.

[plb70288-bib-0036] Jensen W.A. (1962) Botanical histochemistry. WH Freeman, San Francisco.

[plb70288-bib-0037] Jersáková J. , Johnson S.D. , Kindlmann P. (2006) Mechanisms and evolution of deceptive pollination in orchids. Biological Reviews, 81, 219–235. 10.1017/S1464793105006986 16677433

[plb70288-bib-0038] Jia L.B. , Huang S.Q. (2022) An examination of nectar production in 34 species of *dendrobium* indicates that deceptive pollination in the orchids is not popular. Journal of Systematics and Evolution, 60, 1371–1377.

[plb70288-bib-0039] Johansen D.A. (1940) Plant Microtechnique. New York, NY, USA: McGraw‐Hill Book Co, pp 523.

[plb70288-bib-0040] Johnson S.D. , Peter C.I. , Nilsson L.A. , Ågren J. (2003) Pollination success in a deceptive orchid is enhanced by co‐occurring rewarding magnet plants. Ecology, 84, 2919–2927.

[plb70288-bib-0041] Karremans A.P. , Bogarín D. , Otárola M.F. , Sharma J. , Watteyn C. , Warner J. , Herrera B.R. , Chinchilla I.F. , Carman E. , Valerio E.R. , Huarcaya R.P. , Whitworth A. (2023) First evidence for multimodal animal seed dispersal in orchids. Current Biology, 33, 364–371. 10.1016/j.cub.2022.11.041 36521493

[plb70288-bib-0042] Kearns C.A. , Inouye D.W. (1993) Techniques for pollination biologists. Niwot: University Press of Colorado.

[plb70288-bib-0043] Kephart S.R. (1983) The partitioning of pollinators among three species of *Asclepias* . Ecology, 64, 120–133.

[plb70288-bib-0044] Köppen W.P. (1948) Climatología: con un estudio de los climas de la tierra. Fondo de Cultura Económica, Ciudad del México.

[plb70288-bib-0045] Krahl A.H. , Holanda A.S. , Krahl D.R. , Martucci M.E.P. , Gobbo‐Neto L. , Webber C. , Pansarin E. (2019) Study of the reproductive biology of an Amazonian *Heterotaxis* (Orchidaceae) demonstrates the collection of resin‐like material by stingless bees. Plant Systematics and Evolution, 305, 281–291.

[plb70288-bib-0046] Landim C.C. , Stort A.C. , Cruz M.A.C. , Kitajima E.W. (1965) Órgāo Tibial Dos Machos De Euglossini. Estudo Ao Microscópio Óptico E Eletrônico. Revista Brasileira de Biologia, 25, 323.

[plb70288-bib-0048] Liu Y.B. , Zeng Z.J. , Barron A.B. , Ma Y. , He Y.Z. , Liu J.F. , Li Z. , Yan W.Y. , He X.J. (2022) The involvement of a floral scent in plant‐honeybee interaction. The Science of Nature, 109, 30. 10.1007/s00114-022-01802-z 35643865

[plb70288-bib-0050] Magrini S. , Barreca D. , Zucconi L. (2019) A rapid double‐staining technique to improve seed viability testing in terrestrial orchids. Plant Biosystems‐An International Journal Dealing with all Aspects of Plant Biology, 153, 877–882.

[plb70288-bib-0051] Makino T.T. , Ohashi K. (2017) Honest signals to maintain a long‐lasting relationship: floral colour change prevents plant‐level avoidance by experienced pollinators. Functional Ecology, 31, 831–837.

[plb70288-bib-0052] Martel C. , Gerlach G. , Ayasse M. , Milet‐Pinheiro P. (2019) Pollination ecology of the neotropical gesneriad *Gloxinia perennis*: chemical composition and temporal fluctuation of floral perfume. Plant Biology, 21, 723–731. 10.1111/plb.12974 30734450

[plb70288-bib-0053] Martini P. , Schlindwein C. , Montenegro A. (2003) Pollination, flower longevity, and reproductive biology of *Gongora quinquenervis* Ruíz and Pavón (Orchidaceae) in an Atlantic forest fragment of Pernambuco, Brazil. Plant Biology, 5, 495–503.

[plb70288-bib-0116] Martins D.C. , de Albuquerque P.M.C. , Silva F.S. , Rebêlo J.M.M. (2018) Orchid bees (Apidae: Euglossini) in Cerrado remnants in northeast Brazil. Journal of Natural History, 52(11‐12), 627–644. 10.1080/00222933.2018.1444210

[plb70288-bib-0056] Milet‐Pinheiro P. , Domingos‐Melo A. , Olivera J.B. , Albuquerque N.S.L. , Costa A.C.G. , Albuquerque‐Lima S. , Silva M.F.R. , Navarro D.M.A.F. , Maia A.C.D. , Gundersen L.L. , Schubert M. , Dotterl S. , Machado I.C. (2021) A semivolatile floral scent marks the shift to a novel pollination system in bromeliads. Current Biology, 31, 860–868. 10.1016/j.cub.2020.11.012 33338429

[plb70288-bib-0057] Milet‐Pinheiro P. , Navarro D.M.D.A.F. , Dötterl S. , Carvalho A.T. , Pinto C.E. , Ayasse M. , Schlindwein C. (2015) Pollination biology in the dioecious orchid *Catasetum uncatum*: how does floral scent influence the behaviour of pollinators? Phytochemistry, 116, 149–161. 10.1016/j.phytochem.2015.02.027 25771507

[plb70288-bib-0058] Milet‐Pinheiro P. , Pinto C.E. , Navarro D.M. , Silva J.B.F. , Brandt K. , Ayasse M. (2022) Floral scent chemistry and pollinators of a sexually dimorphic neotropical orchid. Plants, 12, 17. 10.3390/plants12010017 PMC9824664 36616146

[plb70288-bib-0059] Milet‐Pinheiro P. , Silva J.B.F. , Navarro D.M. , Machado I.C. , Gerlach G. (2018) Notes on pollination ecology and floral scent chemistry of the rare neotropical orchid *Catasetum galeritum* Rchb. f. Plant Species Biology, 33, 158–163.

[plb70288-bib-0060] Monteiro S.H.N. (2024) Galeandra in Flora e Funga do Brasil. Jardim Botânico do Rio de Janeiro. https://floradobrasil.jbrj.gov.br/FB11597

[plb70288-bib-0061] Monteiro S.H.N. , Selbach‐Schnadelbach A. , Oliveira R.P. , van den Berg C. (2010) Molecular phylogenetics of *Galeandra* (Orchidaceae: Catasetinae) based on plastid and nuclear DNA sequences. Systematic Botany, 35, 476–486.

[plb70288-bib-0065] Nunes C.E. , Peñaflor M.F.G. , Bento J.M.S. , Salvador M.J. , Sazima M. (2016) The dilemma of being a fragrant flower: the major floral volatile attracts pollinators and florivores in the euglossine‐pollinated orchid *Dichaea pendula* . Oecologia, 182, 933–946. 10.1007/s00442-016-3703-5 27538674

[plb70288-bib-0066] Nunes C.E. , Wolowski M. , Pansarin E.R. , Gerlach G. , Aximoff I. , Vereecken N.J. , Salvador M.J. , Sazima M. (2017) More than euglossines: the diverse pollinators and floral scents of Zygopetalinae orchids. The Science of Nature, 104, 1–17. 10.1007/s00114-017-1511-3 29028068

[plb70288-bib-0067] Opedal Ø.H. , Martins A.A. , Marjakangas E.L. (2020) A database and synthesis of euglossine bee assemblages collected at fragrance baits. Apidologie, 51, 519–530.

[plb70288-bib-0069] Pansarin E.R. (2022) Vanilla flowers: much more than food‐deception. Botanical Journal of the Linnean Society, 198, 57–73.

[plb70288-bib-0071] Pansarin E.R. , Ferreira A.W.C. (2022) Evolutionary disruption in the pollination system of *vanilla* (Orchidaceae). Plant Biology, 24, 157–167. 10.1111/plb.13356 34724285

[plb70288-bib-0072] Pansarin E.R. , Maciel A.A. (2017) Evolution of pollination systems involving edible trichomes in orchids. AoB Plants, 9, plx033.

[plb70288-bib-0073] Pansarin L.M. , Castro M.M. , Sazima M. (2009) Osmophore and elaiophores of *Grobya amherstiae* (Catasetinae, Orchidaceae) and their relation to pollination. Botanical Journal of the Linnean Society, 159, 408–415.

[plb70288-bib-0075] Pansarin L.M. , Pansarin E.R. , Sazima M. (2008) Reproductive biology of *Cyrtopodium polyphyllum* (Orchidaceae): a Cyrtopodiinae pollinated by deceit. Plant Biology, 10, 650–659. 10.1111/j.1438-8677.2008.00060.x 18761503

[plb70288-bib-0076] Pansarin L.M. , Pansarin E.R. , Sazima M. (2014) Osmophore structure and phylogeny of Cirrhaea (Orchidaceae, Stanhopeinae). Botanical Journal of the Linnean Society, 176, 369–383.

[plb70288-bib-0078] Pearse A.G.E. (1980) The chemistry and practice of fixation, Histochemistry, theoretical and applied, preparative and optical technology, Vol. 1, Ediburg: Churchill Livingstone, pp 97–158.

[plb70288-bib-0079] Pearson D.L. , Dressler R.L. (1985) Two‐year study of male orchid bee (Hymenoptera: Apidae: Euglossini) attraction to chemical baits in lowland south‐eastern Peru. Journal of Tropical Ecology, 1, 37–54.

[plb70288-bib-0080] Pellegrino G. , Gargano D. , Noce M.E. , Musacchio A. (2005) Reproductive biology and pollinator limitation in a deceptive orchid, *Serapias vomeracea* (Orchidaceae). Plant Species Biology, 20, 33–39.

[plb70288-bib-0081] Pemberton R.W. , Wheeler G.S. (2006) Orchid bees don't need orchids: evidence from the naturalization of an orchid bee in Florida. Ecology, 87, 1995–2001. 10.1890/0012-9658(2006)87[1995:obdnoe]2.0.co;2 16937639

[plb70288-bib-0082] Pérez‐Escobar O.A. , Gottschling M. , Whitten W.M. , Salazar G. , Gerlach G. (2016) Sex and the Catasetinae (Darwin's favourite orchids). Molecular Phylogenetics and Evolution, 97, 1–10. 10.1016/j.ympev.2015.11.019 26708054

[plb70288-bib-0084] Phillips R.D. , Reiter N. , Peakall R. (2020) Orchid conservation: from theory to practice. Annals of Botany, 126, 345–362. 10.1093/aob/mcaa093 PMC7424752 32407498

[plb70288-bib-0085] Pontes C.A.D.S. , Domingos‐Melo A. , Milet‐Pinheiro P. , Navarro D.M.D.A.F. , Nadia T.L. , Machado I.C. (2022) Staminode of *Jacaranda rugosa* AH gentry (Bignoniaceae) promotes functional specialization by ensuring signaling and mechanical fit to medium‐sized bees. Organisms Diversity & Evolution, 22, 527–541.

[plb70288-bib-0117] Raguso R.A. (2004) Flowers as sensory billboards: progress towards an integrated understanding of floral advertisement. Current Opinion in Plant Biology, 7(4), 434–440. 10.1016/j.pbi.2004.05.010 15231267

[plb70288-bib-0086] Ramírez S.R. , Eltz T. , Fujiwara M.K. , Gerlach G. , Goldman‐Huertas B. , Tsutsui N.D. , Pierce N.E. (2011) Asynchronous diversification in a specialized plant‐pollinator mutualism. Science, 333, 1742–1746. 10.1126/science.1209175 21940893

[plb70288-bib-0118] Rech A.R. , Agostini K. , Oliveira P.E. , Machado I.C. (Eds) (2014) Biologia da polinização. Projecto Cultural, Rio de Janeiro, 524.

[plb70288-bib-0087] Reposi S.D. , Gotelli M.M. , Torretta J.P. (2021) Anatomy and ultrastructure floral osmophores of *Catasetum fimbriatum* (Orchidaceae). Protoplasma, 258, 1–12. 10.1007/s00709-021-01625-1 33619655

[plb70288-bib-0088] Reyes H.C. , Draper D. , Marques I. (2021) Pollination in the rainforest: scarce visitors and low effective pollinators limit the fruiting success of tropical orchids. Insects, 12, 856. 10.3390/insects12100856 PMC8538420 34680625

[plb70288-bib-0119] Romero G. , Carnevali G. , Pridgeon A.M. (2009) Catasetinae. Genera Orchidacearum, Epidendroideae, 5, 13–18.

[plb70288-bib-0091] Santos I.S.D. , Silva M.J.D. (2020) The genus *Galeandra* Lindl. (Orchidaceae, Epidendroideae) in the Federal District and the state of Goiás, Brazil. Hoehnea, 47, e462020.

[plb70288-bib-0092] Sazima M. , Vogel S. , Cocucci A. , Hausner G. (1993) The perfume flowers of *Cyphomandra* (Solanaceae): pollination by euglossine bees, bellows mechanism, osmophores, and volatiles. Plant Systematics and Evolution, 187, 51–88.

[plb70288-bib-0120] Schiestl F.P. (2005) On the success of a swindle: pollination by deception in orchids. Naturwissenschaften, 92(6), 255–264. 10.1007/s00114-005-0636-y 15931514

[plb70288-bib-0095] Stern W.L. , Curry K.J. , Pridgeon A.M. (1987) Osmophores of *Stanhopea* (Orchidaceae). American Journal of Botany, 74, 1323–1331.

[plb70288-bib-0096] Suetsugu K. , Naito R.S. , Fukushima S. , Kawakita A. , Kato M. (2015) Pollination system and the effect of inflorescence size on fruit set in the deceptive orchid *Cephalanthera falcata* . Journal of Plant Research, 128, 585–594. 10.1007/s10265-015-0716-9 25801274

[plb70288-bib-0097] Vogel S. (1963) Duftdrüsen im Dienste der Bestäubung: èber Bau und Funktion der Osmophoren. Mainz, Germany: Akademie der Wissenschaften und der Literatur.

[plb70288-bib-0099] Vogel S. (1990) The role of scent glands in pollination: on the structure and function of osmophores. Amerind, New Delhi, India.

[plb70288-bib-0100] Vudala S.M. , Ribas L.L.F. (2017) Seed storage and asymbiotic germination of *Hadrolaelia grandis* (Orchidaceae). South African Journal of Botany, 108, 1–7.

[plb70288-bib-0101] Watteyn C. , Scaccabarozzi D. , Muys B. , Reubens B. , Ackerman J.D. , Otárola M.F. , Amador M.F.G. , Karremans A.P. (2023) Sweet as *Vanilla hartii*: evidence for a nectar‐rewarding pollination mechanism in *vanilla* (Orchidaceae) flowers. Flora, 303, 152294.

[plb70288-bib-0102] Watteyn C. , Scaccabarozzi D. , Muys B. , Schueren N.C.D. , Meerbeek K.V. , Amador M.F.G. , Ackerman J.D. , Fonseca M.V.C. , Alvadaro I.F.C. , Reubens B. , Huarcaya R.P. , Cozzolino S. (2022) Trick or treat? Pollinator attraction in *Vanilla pompona* (Orchidaceae). Biotropica, 54, 268–274.

[plb70288-bib-0104] Whitten W.M. , Young A.M. , Stern L. (1993) Nonfloral sources of chemicals that attract male euglossine bees (Apidae: Euglossini). Journal of Chemical Ecology, 19, 3017–3027. 10.1007/BF00980599 24248792

[plb70288-bib-0105] Wickham H. (2016) *ggplot2*: elegant graphics for data analysis. Springer‐Verlag, New York.

[plb70288-bib-0106] Williams N.H. , Whitten W.M. (1983) Orchid floral fragrances and male euglossine bees: methods and advances in the last sesquidecade. The Biological Bulletin, 164, 355–395.

