# Supplementary material for: A dual pollination strategy combining perfume attraction and food deception in Galeandra blanchetii E.S. Rand (Orchidaceae)
Source: Plant Biol (Stuttg). 2026 Sep 25;28(7):2313–26. doi: 10.1111/plb.70288 (PMC13750324; doi:10.1111/plb.70288)
Supplement: Supplementary file 1 [file PLB-28-2313-s001.docx]

Scent, deception, or both? Unraveling the enigma of the pollination mechanism of Galeandra blanchetii E.S. Rand (Orchidaceae)

Deborah Maciel Camara^1,*^, Alessandro Wagner Coelho Ferreira^2^, Ana Paula Souza Caetano^3^, Arthur Domingos-Melo^4^, Sinzinando Albuquerque-Lima^5^; Paulo Milet-Pinheiro^6^, Isabel Cristina Machado^7^, Edlley Max Pessoa^8^

SUPPLEMENTARY TABLES

| **Table S1.** Summary of observations of *Galeandra blanchetii* (Orchidaceae) carried out on the field in two study areas in São Luís, Maranhão, Brazil. *UFMA: Universidade Federal do Maranhão; APA Itapiracó: Environmental Protection Area Itapiracó. | | | | |
| --- | --- | --- | --- | --- |
| Species | Date | Duration | Location | Hours |
| *G. blanchetii* | 23-25 Jun 2018 | 07h00–12h00 | UFMA | 15 h |
|  | 01-15 Jul 2018 | 07h00–12h00 | UFMA | 75 h |
|  | 03-05 Aug 2018 | 07h00–12h00 | UFMA | 15 h |
|  | 03-09 Apr 2019 | 07h00–12h00 | UFMA | 35 h |
|  | 01-05 May 2019 | 13h00-17h00 | UFMA | 20 h |
|  | 23-25 Feb 2021 | 07h00-12h00 | APA ITAPIRACÓ | 15 h |
|  | 13-15 May 2022 | 06h00-17h00 | UFMA | 30 h |
|  | 06-08 Jun 2022 | 06h00-17h00 | UFMA | 30 h |
|  | 10-24 Jul 2022 | 06h00-17h00 | UFMA | 150 h |
|  | 06-08 Mar 2023 | 06h00-17h00 | UFMA | 30 h |
|  | 13-17 Apr 2023 | 06h00-17h00 | UFMA | 50 h |
|  | 09-13 May 2023 | 06h00-17h00 | UFMA | 50 h |
| Total hours: |  |  |  | 515 hours |

| **Table S2**. Morphological measures of *Galeandra blanchetii* (Orchidaceae) in populations in São Luís, Maranhão, Brazil. | | | | | | |
| --- | --- | --- | --- | --- | --- | --- |
| ***G. blanchetii*** | | | | | | |
| Sepals | | Petals | Labellum | Spur | Column | Pollinarium |
| Dorsal | Lateral |  |  |  |  |  |
| 25–26 ×  5.1–5.5mm | 25–27 ×  5.2–5.3 mm | 21–26.9  × 5.3–5.5 mm | 56–68 ×  13–15.7 mm | 10.07–13.87mm | 11–12 ×  5–5.2 mm | 1.4–1.5 ×  1.6–1.7 mm |

| **Table S3**. Histochemical analysis of flowers of *Galeandra blanchetii* (Orchidaceae)*.* | | | | | |
| --- | --- | --- | --- | --- | --- |
| **Test** | **Target compound** | **Reference** | **Result** | | |
|  |  |  | **LB_ad_** | **LB_ab_** | **CC** |
| Ruthenium red | Pectin/Mucilage | Gregory & Baas, 1989 | – | – | – |
| Lugol | Starch | Jensen, 1962 | + | – | – |
| Sudan IV | Total lipids | Pearse,1980 | + | + | – |
| NADI reagent | Terpenes | David & Carde, 1964 | + | + | – |
| Coomassie brilliant blue | Proteins | Fisher, 1968 | – | – | – |
| Periodic acid-Schiff (PAS) | Polysaccharides (insoluble) | Feder & O'Brien, 1968 | – | – | – |
| Ferric chloride | Phenolic compounds | Johansen, 1940 | – | – | – |

(+) positive reaction; (–) negative reaction.

| **Table S4.** Percentage of *Euglossa* bees observed (including not collected) in relation to the frequency of search for nectar or scent in *Galeandra blanchetii* (Orchidaceae) in São Luís, Maranhão, Brazil. | | |
| --- | --- | --- |
| **Behavior** | ***Euglossa* spp*.* (male)**  **(*N* = 19)** | ***Euglossa* spp*.* (female)**  **(*N* = 14)** |
| Scent search | 3 (15.78%) | - |
| Nectar search | 16 (84.22%) | 14 (100%) |

| **Table S5.** Measures of *Galeandra blanchetii* (Orchidaceae) flowers (N=30). AN: Height from the labellum up to the mean anther region; CN: Height from the labellum to the rostellum cup; LT: Floral tube width; and CC: Spur lenght. | | | | |
| --- | --- | --- | --- | --- |
| **Species** | **Measurements** | | | |
|  | **AN** | **CN** | **LT** | **CC** |
| *G. blanchetii* | 5.6±0.86 | 4.6±0.81 | 7.4±1.40 | 12.9±1.77 |

| **Table S6.** Measurements performed on bees collected in *Galeandra blanchetii* (Orchidaceae) flowers, in natural populations in Maranhão, Brazil. | | | |
| --- | --- | --- | --- |
|  | **Thorax height**  **(mean ± *SD*)** | **Intertegular distance**  **(mean ± SD)** | **Glossa length**  **(mean ± *SD*)** |
| ***Euglossa* sp1** (♀) | 4.1 ± 0.29 | 4.4 ± 0.34 | 7.4 ± 0.59 |
| ***Euglossa amazonica*** | 4.21 | 4.29 | 8.68 |
| ***Euglossa cordata*** | 4.5 ± 0.22 | 4.6 ± 0.19 | 7.6 ± 0.78 |
| ***Euglossa securigera*** | 4.4 ± 0.16 | 4.6 ± 0.14 | 8.3 ± 1.06 |
| ***Eulaema nigrita*** | 7.6 | 7.2 | 11.23 |
| ***Ceratina*** | 1.9 | 2.1 | 3.2 |
| ***Trigona*** | 2.5 | 2.0 | 1.8 |

| **Table S7.** Result of fruit formation in *Galendra blanchetii* (Orchidaceae) according to experimental treatments and natural conditions in plants from two study locations in São Luís, in the state of Maranhão, Brazil. | | | |
| --- | --- | --- | --- |
| **Treatment** | **Flower number** | **Fruit set (%)** | **Embryo viability (%)** |
| 2021 | | | |
| Emasculation | 30 | – | – |
| Spontaneous self-pollination | 30 | – | – |
| Natural pollination | 42 | 8 (19.04%) | – |
| 2022 | | | |
| Self-pollination | 30 | 30 (100%) | 68.32% (6.000) |
| Crossed pollination | 30 | 30 (100%) | 94.22% (6.000) |
| Natural pollination | 46 | 8 (17.39%) | 95.75% (1.600) |
